# Evaluation of Bioactive Compounds and Antioxidative Activity of Fermented Green Tea Produced via One- and Two-Step Fermentation

**DOI:** 10.3390/antiox11081425

**Published:** 2022-07-22

**Authors:** Huiling Xu, Jong Hyoung Hong, Dabin Kim, Young Hun Jin, Alixander Mattay Pawluk, Jae-Hyung Mah

**Affiliations:** Department of Food and Biotechnology, Korea University, Sejong 30019, Korea; huiling_xu@korea.ac.kr (H.X.); hjh0621@korea.ac.kr (J.H.H.); karl4567@korea.ac.kr (D.K.); younghoonjin3090@korea.ac.kr (Y.H.J.); alixei@korea.ac.kr (A.M.P.)

**Keywords:** fermented green tea, one- and two-step fermentation, acetic acid fermentation, lactic acid fermentation, fermentation optimization, *Acetobacter pasteurianus*, organic acids, γ-aminobutyric acid, DPPH scavenging activity, polyphenols

## Abstract

This study investigated the influence of one- and two-step fermentation on bioactive compound production in fermented green tea, i.e., one-step fermented green tea (OFG) and two-step fermented green tea (TFG). One-step fermentation entailed acetic acid fermentation, while two-step fermentation consisted of lactic acid fermentation followed by acetic acid fermentation. *Acetobacter pasteurianus* PCH 325, isolated from an over-ripened peach, was selected for acetic acid fermentation based on its growth and organic acid production characteristics. Acetic acid fermentation conditions were optimized for one- and two-step fermentation: 3% fermentation alcohol for both processes; 8% and 4% sucrose, respectively; and fermentation at 25 °C for both processes. For lactic acid fermentation of TFG, the inoculum and optimized conditions reported previously were used. Under the optimized conditions, the acetic acid content in OFG and TFG increased 21.20- and 29.51-fold, respectively. Furthermore, through two-step fermentation, γ-aminobutyric acid and lactic acid were produced up to 31.49 ± 1.17 mg/L and 243.44 ± 58.15 mg/L, respectively, which together with acetic acid could contribute to the higher DPPH scavenging activity of TFG. This study suggests that two-step fermentation may be a valuable strategy in industry for raising the amount of acetic acid and/or providing additional bioactive compounds.

## 1. Introduction

Tea, brewed from *Camellia sinensis* leaves [1], is a beverage classified based on oxidation level—black tea (fully oxidized leaves), oolong tea (semi-oxidized leaves), and green tea (unoxidized leaves) [2]. Particularly, green tea has been enjoyed globally for over 800 years, and contains many bioactive compounds, including polyphenols (e.g., catechins, flavanols, flavandiols, and phenolic acids), caffeine, theanine, chlorophyll, and vitamins [3,4,5,6,7]. These compounds have been reported to provide antioxidative, anti-obesity, and anti-carcinogenic effects [3,4,5,6,7].

Fermentation is one of the oldest and most important technologies for the production and preservation of foods [8,9,10]. Recently, fermentation has garnered attention as a technology for enhancing the functionality and organoleptic properties of foods [11]. The health-promoting effects of green tea can likely be improved by fermentation and/or biotransformation. Particularly, lactic acid bacteria (LAB) are known to degrade green tea’s catechins and polyphenols into smaller phenolic acids and other metabolites, which can be more antioxidative and bioavailable than the original substrates [12]. For instance, Wang et al. [13] reported that lactic acid fermentation using *Lacticaseibacillus paracasei* (formerly *Lactobacillus paracasei*) enhanced the anti-obesity effect of green tea. Jin et al. [14] also reported that the use of LAB increased the amounts of a variety of bioactive compounds with health-promoting effects in green tea. In the meantime, acetic acid bacteria (AAB)—such as *Acetobacter pasteurianus*, *A. aceti*, and *A. lovaniensis*—have been extensively utilized in the acetic acid fermentation of various food materials. AAB can efficiently convert ethanol to acetic acid, which is why they are of importance in the vinegar industry [15,16]. Acetic acid has been extensively reported to have antimicrobial [17], antioxidative [18], anti-hyperglycemic [19], antidiabetic [20], anti-hypertensive [21], hypocholesterolemic [22], anti-obesity [23], and anti-carcinogenic [24] effects. Therefore, organic vinegars produced through acetic acid fermentation of various fermentation materials have been developed and consumed worldwide [25]. Intensive research on the optimization of key fermentation factors—such as fermentation alcohol, carbohydrates, and fermentation temperature—to stimulate acetic acid production by AAB has also been conducted [26,27,28]. However, research on the acetic acid fermentation of green tea is limited.

Several previous studies have tried to develop two-step fermentation processes to enhance and/or provide health-promoting effects of existing or novel bioactive compounds compared to one-step fermentation. Liu et al. [29] reported that two-step fermentation using *Lactobacillus rhamnoides* and *Bacillus amyloliquefaciens* improved the nutritional value of shrimp shell. Luan et al. [30] used *Saccharomyces cerevisiae* and mixed LAB strains to produce two-step-fermented horseradish sauce with higher nutritional quality and superior taste compared to the unfermented sauce. Meanwhile, Horiuchi et al. [31] reported that two-step fermentation of onion vinegar using *S. cerevisiae* and *A. pasteurianus* significantly increased the content of acetic acid. The use of AAB, however, has rarely been employed for two-step fermentation along with LAB. Moreover, to the best of our knowledge, studies on the impact of acetic acid fermentation on the functionality of green tea and the two-step fermentation of green tea are rarely found in the literature.

As such, the present study evaluated the effect of two-step fermentation—consisting of lactic acid fermentation followed by acetic acid fermentation—on the functionality of fermented green tea, compared to one-step fermentation. In detail, the research was carried out to stimulate acetic acid fermentation (as one-step acetic acid fermentation, or as part of two-step acetic acid fermentation) of green tea, while focusing on the selection of AAB strains with outstanding acetic acid production isolated from over-ripened fruits, along with the optimization of fermentation factors. Moreover, this study investigated the influence of one-step fermentation (i.e., acetic acid fermentation) with the selected strain—*A. pasteurianus* PCH 325—and two-step fermentation (i.e., lactic acid fermentation followed by acetic acid fermentation) with a prolific γ-aminobutyric acid (GABA)-producing strain—*Levilactobacillus brevis* GTL 79—and successively with *A. pasteurianus* PCH 325, under optimized conditions, on the production of bioactive compounds in one-step fermented green tea (OFG) and two-step fermented green tea (TFG). Finally, the antioxidative activity of both OFG and TFG was evaluated, and the contribution of bioactive compounds produced by the fermentation processes to the activity was also discussed.

## 2. Materials and Methods

### 2.1. Acetobacter Strains Used

#### 2.1.1. Isolation of Acetobacter Strains from Over-Ripened Fruits

Isolation of *Acetobacter* strains was conducted as described in a previous report [32], with slight modification. Samples of over-ripened fruits—including apples, peaches, apricots, and plums—were collected from market vendors in Sejong, Republic of Korea, and stored at 4 °C. Within 24 h of storage, *Acetobacter* strains were isolated from the samples. Three grams of each over-ripened fruit was homogenized with 30 mL of 0.1% peptone saline in a stomacher (Laboratory Blender Stomacher 400, Seward, Ltd., Worthing, UK). Three milliliters of the homogenate was then combined with 7 mL of enrichment medium I (1.5% peptone, 0.8% yeast extract, 1% glucose, 0.5% ethanol, 0.3% acetic acid, 0.01% cycloheximide; adjusted to a pH of 3.5 using 2 M hydrochloric acid; all from Junsei Chemical Co., Tokyo, Japan). After incubation at 30 °C for 72 h, the medium was serially diluted with 0.1% peptone saline. One hundred microliters of each dilution was spread-inoculated in duplicate on calcium carbonate agar (2% glucose, 0.8% yeast extract, 0.7% calcium carbonate, 0.5% ethanol, 0.5% peptone, 1.2% agar; all from Junsei). After aerobic incubation at 30 °C for 72 h, colonies with a clear zone—indicating AAB—formed on agar plates containing 10–300 colonies [33] were streak-inoculated onto YPM agar (0.5% yeast extract, 0.3% peptone, 2.5% mannitol, 1.2% agar; all from Junsei) and incubated under the same conditions to differentiate individual strains. To obtain pure cultures, single colonies were streak-inoculated on YPM agar and incubated under the same conditions. Then, the single colonies were picked and inoculated into 5 mL of YPM broth and aerobically incubated at 30 °C for 72 h. The culture was stored at −20 °C as a 20% glycerol stock solution (*v*/*v*).

#### 2.1.2. Identification of the Selected Acetobacter Strain

*Acetobacter* strains were characterized and selected on the basis of their capability of oxidizing acetate [34]. The selected *Acetobacter* strains were further identified using 16S rRNA gene sequence analysis. The universal bacterial primer pair 518F (5′-CCAGCAGCCGCGGTAATACG-3′) and 805R (5′-GACTACCAGGGTATCTAAT-3′) (all from Solgent Co., Daejeon, Korea) was used for the amplification of the 16S rRNA gene. The identities of sequences were determined using the basic local alignment search tool (BLAST) of the National Center for Biotechnology Information [35]. Finally, the strain identified as *A. pasteurianus* PCH 325 was used for acetic acid fermentation of green tea.

#### 2.1.3. Reference Strain Used

*Acetobacter aceti* KCTC 12290 (also designated as ATCC 15973), obtained from the Korean Collection for Type Cultures (KCTC; Daejeon, Korea), was utilized as a reference strain to compare the growth and acid production characteristics of the isolated AAB strains, as it is one of the most widely used strains in the industrial production of vinegar [36].

### 2.2. Acetobacter Strain Selection for Acetic Acid Fermentation of OFG and TFG

**Figure 1:**
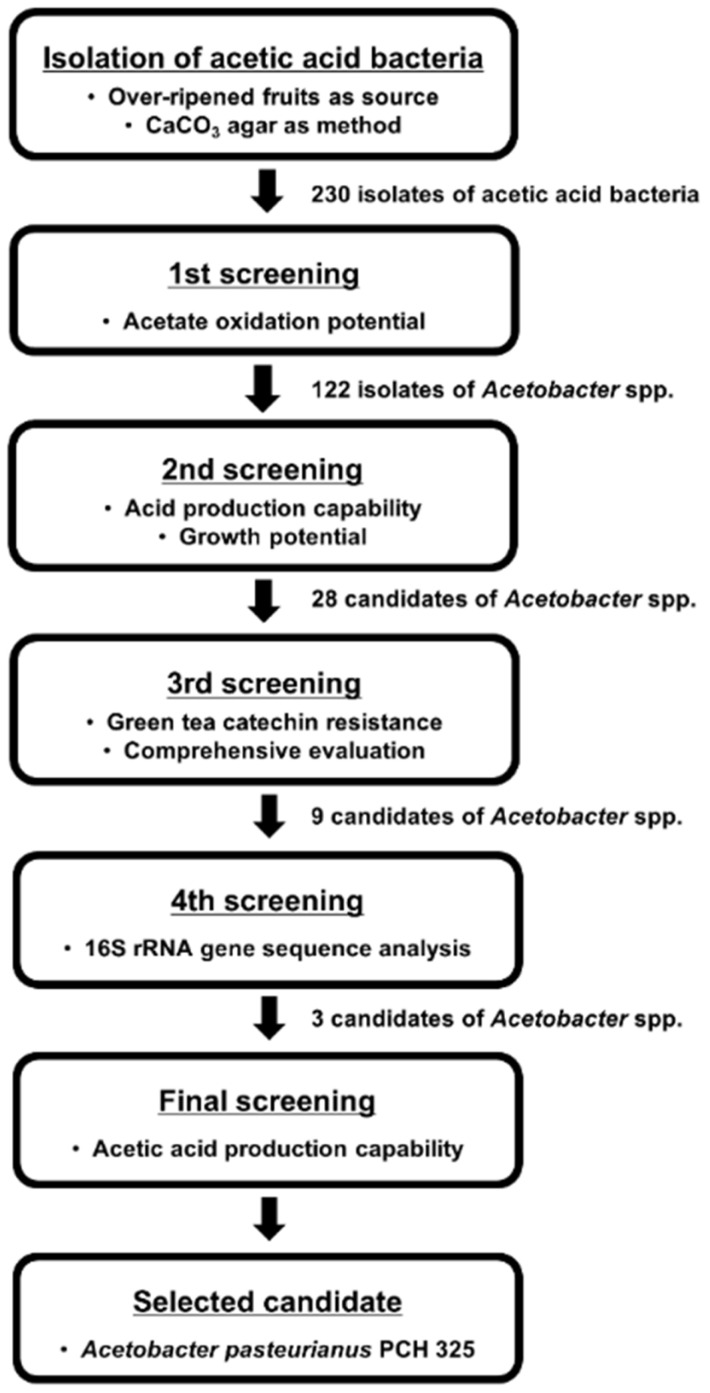
Flowchart of the screening process for selecting the strain used for acetic acid fermentation of green tea.

The isolated *Acetobacter* strain ultimately used in the acetic acid fermentation of OFG and TFG was selected on the basis of its acid production capability and growth potential. Resistance to green tea catechins and acetic acid-producing capability were also considered in strain selection. Five milliliters of YPM broth was inoculated with approximately 10 μL of glycerol stock of each AAB strain (either reference or each isolated strain). After aerobic incubation at 30 °C for 72 h, 100 μL of the culture was inoculated into 5 mL of fresh YPM broth and incubated under the same conditions. This culture was used for the 4 different tests (i.e., acid production capability, growth potential, resistance to green tea catechins, and acetic acid-producing capability) of the screening process (Figure 1). The culture was also used for the preparation of the bacterial suspension (see Section 2.3), which was used in the fourth screening test (Section 2.2) as well as the acetic acid fermentation of green tea (see Section 2.6).

The first 3 tests followed methods described by Jin et al. [14], with minor modifications, including basal medium (1% peptone, 1% glucose, 1% ethanol, 1% glycerol, 0.5% yeast extract; adjusted to a final pH of 6.8 by adding 2 M hydrochloric acid) [32] for the acid production capability test and YPM broth for the growth potential and resistance to green tea catechins tests, with incubation at 30 °C for 72 h to obtain the strains with the highest acid production and growth potential in the presence of fermentation alcohol [37]. Specific growth rates and lag times were calculated based on the values generated by Bioscreen C (Labsystems, Helsinki, Finland), using spreadsheet software (Excel 2016; Microsoft Co., Redmond, WA, USA) and a mathematical model developed by Baranyi and Roberts [38]. The relative lag time was calculated based on previous studies [39,40,41], with slight modifications. The relative lag time is the ratio of the lag time (h) of the tested AAB strains to the value of the strain with the shortest lag time. On the basis of this formula, a value of 1.00 indicates the shortest lag time.

The acetic acid-producing capability (fourth screening test) of the *Acetobacter* strains was measured by ion chromatography analysis (see Section 2.8) of test samples prepared by inoculating 200 mL of green tea (see Section 2.4) with bacterial suspension (see Section 2.3) at a concentration of 6 Log CFU/mL, followed by aerobic fermentation at 30 °C for 6 days.

### 2.3. Bacterial Suspension Preparation for Acetic Acid Fermentation of Green Tea

The suspension was prepared following a method described previously [14], with slight modification. In detail, 250 mL of YPM broth was inoculated with 5 mL of the culture prepared above, and incubated aerobically at 30 °C for 72 h. The culture was then centrifuged at 8000× *g* and 4 °C for 5 min. After washing three times, the pellet was resuspended in a buffer (Sörensen’s buffer; 5.675 g of disodium phosphate, 3.630 g of monopotassium phosphate). The suspension (adjusted to 8 Log CFU/mL) was used in the acetic acid fermentation of green tea (see Section 2.2 and Section 2.6.1).

### 2.4. Green Tea Preparation

The green tea was brewed as previously described [14]. Eighty grams of dried leaves was infused for 5 min in 2 L of boiling distilled water. The infused green tea was then filtered through a sterile sieve. The filtrate was used in one-step fermentation and in the lactic acid fermentation phase of two-step fermentation.

### 2.5. Lactic Acid-Fermented Green Tea Preparation for Two-Step Fermentation

Lactic acid-fermented green tea for lactic acid fermentation (the first phase of two-step fermentation) was prepared following the previously optimized fermentation conditions described by Jin et al. [14]. Briefly, green tea (as prepared above) was supplemented with fermentation alcohol (1%) and glucose (6%), inoculated with a prolific GABA-producing *L. brevis* GTL 79, and fermented for 5 days at 37 °C.

To remove LAB, a 0.2 µm membrane (cellulose nitrate; Cytiva, Buckinghamshire, UK) was used to filter the lactic acid-fermented green tea. The resulting lactic acid-fermented green tea was used directly in the acetic acid fermentation phase of two-step fermentation.

### 2.6. Optimization of Key Factors for Acetic Acid Fermentation of Green Tea with A. pasteurianus PCH 325

**Figure 2:**
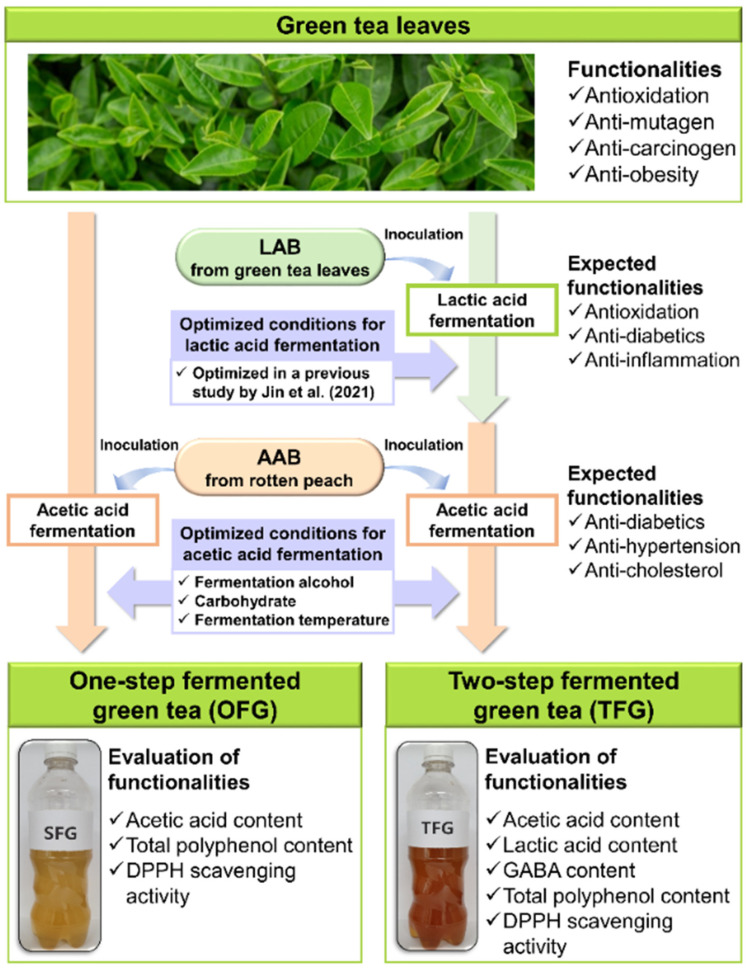
Conceptual scheme of one- and two-step acetic acid fermentation of green tea under optimized fermentation conditions.

As one of the successive optimization processes suggested previously [14,42,43], the optimization process in this study (Figure 2) followed the adaptive one-factor-at-a-time (OFAT) method used in a previous report [14]. Briefly, the key factors for acetic acid fermentation of green tea with *A. pasteurianus* PCH 325—fermentation alcohol, carbohydrates, and fermentation temperature—were optimized for one-step and two-step fermentation. pH, AAB counts, and acetic acid content served as indicators of appropriate acetic acid fermentation. Green tea (see Section 2.4) and lactic acid-fermented green tea (see Section 2.5) were used as the fermentation materials in acetic acid fermentation for each of the above fermentation processes, respectively.

#### 2.6.1. Optimization of Fermentation Alcohol Concentration

To optimize the fermentation alcohol concentration used in one-step fermentation and in the acetic acid fermentation phase of two-step fermentation, fermentation alcohol (95% ethanol, food grade; Ethanol Sales World Co. Ltd., Jeonju, Korea) at a concentration of 0, 1, 3, or 5% (*v*/*v*) was supplemented into 200 mL of the green tea (Section 2.4) or the lactic acid-fermented green tea (Section 2.5) filtrates in an Erlenmeyer flask (500 mL in volume) with a porous silicon stopper. The filtrates were inoculated with the *A. pasteurianus PCH 325* suspension prepared in Section 2.3 to concentrations of approximately 6 Log CFU/mL. The flask was plugged and fermented aerobically at 30 °C for 6 days. The pH, AAB counts, and acetic acid content in OFG and TFG, along with the GABA content and lactic acid content in TFG, were determined at 48 h intervals during fermentation.

#### 2.6.2. Optimization of Carbohydrate Type and Concentration

To optimize the carbohydrate type used in one-step fermentation and in the acetic acid fermentation phase of two-step fermentation, 10% (*w*/*v*) of each carbohydrate (glucose, fructose, or sucrose; all from Junsei) was dissolved in 200 mL of green tea or lactic acid-fermented green tea along with 3% fermentation alcohol (*v*/*v*; as optimized in Section 3.2 and Section 3.3). Inoculation, fermentation, and sampling were performed as described in Section 2.6.1.

Following optimization of the carbohydrate type, the concentration of sucrose (see Section 3.2 and Section 3.3) supplemented into the green tea or lactic acid-fermented green tea samples was further optimized by testing supplement concentrations of 0, 2, 4, 6, 8, and 10% (*w*/*v*).

#### 2.6.3. Optimization of Fermentation Temperature

To optimize the fermentation temperature used in one-step fermentation and in the acetic acid fermentation phase of two-step fermentation, various fermentation temperatures were tested. Green tea and lactic acid-fermented green tea supplemented with 3% fermentation alcohol and 8% or 4% sucrose (one-step or two-step fermentation, respectively), as optimized in Section 3.2 and Section 3.3, were inoculated as described in Section 2.6.1 and fermented at 20, 25, or 30 °C for 6 days.

### 2.7. Determination of Physicochemical and Microbial Characteristics

The pH and acetic bacterial counts of the OFG and TFG samples were determined as described in a previous study by Jin et al. [14], with minor modifications, including YPM agar and incubation at 30 °C for 72 h for the enumeration of acetic acid bacteria.

### 2.8. Analysis of Contents of Organic Acids and GABA

The acetic acid and lactic acid contents in OFG and TFG were analyzed as described in a previous study [14]. The analysis procedures included the filtration of fermented green tea and chromatographic separation of organic acids.

GABA in TFG was quantified according to a procedure described previously [14]. The analysis procedures included the extraction, derivatization, and chromatographic separation of GABA.

### 2.9. Analysis of Contents of Fermentation Alcohol and Carbohydrates

The fermentation alcohol content in OFG and TFG was determined according to the procedures developed by Crowell and Ough [44], with slight modifications. First, 2 mL of OFG or TFG was mixed with 18 mL of distilled water. Then, 1 mL of the mixture was added to 1 mL of 0.2 M dichromate solution (dissolved in 20% sulfuric acid solution) and incubated at 60 °C for 1 h in a water bath. After cooling at room temperature for 20 min, the absorbance of the mixture was measured at 600 nm with a spectrophotometer (Lambda 35, PerkinElmer Ltd., Waltham, MA, USA). Fermentation alcohol was used as a standard for the calibration curve.

The glucose and sucrose contents in OFG and TFG were analyzed using the Sucrose/D-Fructose/D-Glucose Assay Kit (NZYTech, Lisbon, Portugal). The analysis was conducted according to the manufacturer’s instructions.

### 2.10. Analysis of Antioxidative Activity

The total polyphenol content and DPPH (1,1-diphenyl-2-picrylhydrazyl) radical scavenging activity of OFG and TFG—the most representative indicators for presuming the antioxidative activity of plant extracts, as suggested by Clarke et al. [45] and Chaves et al. [46]—were measured to evaluate the antioxidative activity. Both analyses were conducted as described in a previous study [14].

### 2.11. Statistical Analysis

Data were presented as the means and standard deviations of triplicate results. The significance of differences was assessed by one-way analysis of variance (ANOVA) using Fisher’s multiple comparison module of the Minitab software, version 17 (Minitab, LLC., State College, PA, USA), and differences with probability *p* < 0.05 were deemed statistically significant.

## 3. Results

### 3.1. Acetobacter Strain Selection for Acetic Acid Fermentation of Both OFG and TFG

To enhance acetic acid production in OFG and TFG via acetic acid fermentation with prolific acetic acid-producing AAB, the most active candidate was selected based on the following screening process (Figure 1). In the first screening stage, of the 230 isolated strains, 122 strains that could oxidize acetate were selected as tentative *Acetobacter* species. Next, 28 strains were selected due to having either lower pH (i.e., higher acid production capability) or higher OD_600_ (i.e., higher growth potential) than the reference strain (Table 1). Meanwhile, catechins present in green tea have been described to inhibit microbial growth [47]. Therefore, in the third screening stage, the 28 strains were subjected to a test for resistance to green tea catechins, and all of the strains exhibited better growth potential—e.g., higher specific growth rate and/or shorter relative lag time—than the reference strain. This result indicated that the strains could grow in green tea while resisting green tea catechins. After comprehensive consideration of the growth characteristics, including the catechin resistance and microbial metabolic activity tested above, nine strains were further screened. In the fourth screening stage, the nine strains were identified as *A. pasteurianus* (1 strain), *A. lovaniensis* (2 strains), and *A. okinawensis* (6 strains) based on 16S rRNA sequencing analysis. As *A. pasteurianus* and *A. lovaniensis* are widely used for vinegar production in the food industry [15,16,48], in the final screening stage, these three strains were subjected to a green tea fermentation test to compare their acetic acid production capability in green tea. Compared to the acetic acid-producing capability of the reference strain (0.85 ± 0.25 mg/L) and the other two candidates (0.57 ± 0.44 mg/L), *A. pasteurianus* PCH 325 showed the most outstanding acetic acid production (282.98 ± 25.01 mg/L), and was consequently chosen for acetic acid fermentation. As compiled in Table 1, the ultimately selected strain (*A. pasteurianus* PCH 325) exhibited greater (or similar) metabolic activity and resistance to green tea catechins, and the highest acetic acid-producing capability compared to the reference strain and other isolated *Acetobacter* strains.

### 3.2. Optimization of Key Factors for Fermentation of OFG

For the acetic acid fermentation of both OFG (Section 3.2) and TFG (see Section 3.3), *A. pasteurianus* PCH 325, as selected above, was used as the inoculum. To stimulate the metabolic activity of the selected strain, key fermentation factors were optimized, based on the adaptive OFAT method. In each optimization stage, pH and AAB counts were determined to assess appropriate fermentation. The content of the organic acid was also analyzed concurrently, as acetic acid is one of the most important bioactive compounds produced by AAB.

**Figure 3:**
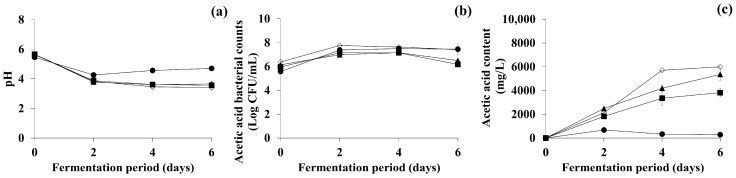
Changes in (**a**) pH, (**b**) acetic acid bacterial counts, and (**c**) acetic acid content in OFG at each stage of the optimization process for acetic acid fermentation. ●: Control (fermentation without supplementation), ■: condition optimized at stage 1 (fermentation with supplementation of 3% fermentation alcohol), ▲: condition optimized at stage 2 (fermentation with supplementation of 8% sucrose in addition to the condition optimized at stage 1), ◇: condition optimized at stage 3 (fermentation at 25 °C in addition to the conditions optimized at stage 2). Sucrose was selected based on the effects of several carbohydrates at the same concentration, and the sucrose concentration was subsequently optimized. Error bars indicate standard deviations determined from triplicate experiments.

Fermentation alcohol is mainly produced by fermentation of grains, such as corn, wheat, and sorghum [49], and has been widely used to promote acetic acid fermentation by AAB in the vinegar industry [50], while concentrations of over 5% may inhibit microbial growth [51]. As such, the present study examined the effects of different fermentation alcohol concentrations (1, 3, and 5%) to define the optimal concentration supplemented into OFG. A control OFG sample without fermentation alcohol supplementation was tested concurrently. Although the change patterns for pH and AAB counts of the control and all supplemented OFG samples were similar (data not shown), the OFG samples with 3% fermentation alcohol supplementation contained a significantly higher level of acetic acid (3818.18 ± 239.56 mg/L) than those of the control and the other supplemented samples (*p* < 0.05) after the 6 days of fermentation. Hence, as shown in Figure 3, 3% fermentation alcohol supplementation was determined to be the optimal factor for acetic acid fermentation in this stage (Figure 3, ■).

The carbohydrate type and concentration can impact AAB growth and metabolic activity [52,53]. As such, in the second optimization stage, OFG samples were supplemented with one of each carbohydrate at a concentration of 10%, along with 3% fermentation alcohol as determined in optimization stage 1. Subsequently, based on the results for pH, AAB counts, and acetic acid production, the best carbohydrate was chosen, and the optimization was rerun with supplementation of 2, 4, 6, 8, or 10% of the carbohydrate to optimize the factor further. A control OFG sample without carbohydrate supplementation was tested concurrently. OFG samples supplemented with sucrose had a relatively lower pH and higher AAB counts as well as significantly higher acetic acid content than the samples supplemented with glucose or fructose (*p* < 0.05) (data not shown). Therefore, sucrose was chosen as the optimal carbohydrate, and the effects of various supplementary concentrations (2, 4, 6, 8, and 10%) were subsequently compared (data not shown). The change patterns for pH and AAB counts of the control and all supplemented OFG samples were similar (data not shown). However, at the end of fermentation, the sucrose-supplemented samples had higher acetic acid concentrations than the control, with 8% sucrose supplementation exhibiting the highest acetic acid-producing capability (5362.54 ± 370.70 mg/L) among all of the samples (*p* < 0.05). Thus, 8% sucrose supplementation was eventually selected as the optimal fermentation factor in optimization stage 2 (Figure 3, ▲).

Fermentation temperature can also influence AAB growth and metabolic activity [52,53]. As such, considering the optimal growth temperature, the present study tested different fermentation temperatures (20, 25, and 30 °C) in the final stage of optimization, along with 3% fermentation alcohol and 8% sucrose supplementation as selected in the earlier stages. The change patterns for pH and AAB counts of all OFG samples fermented at different temperatures were similar (data not shown). However, after fermentation, the OFG sample fermented at 25 °C contained a considerably higher amount of acetic acid (5997.80 ± 513.06 mg/L) than the other samples (*p* < 0.05). Therefore, fermentation at 25 °C was determined to be the optimal factor in this stage (Figure 3, ◇). The changes in pH, acetic acid bacterial count, and acetic acid content obtained under the optimal conditions selected in each optimization stage are compiled in Figure 3. Taken together, for the acetic acid fermentation of OFG, the optimal conditions were determined to be supplementation with 3% fermentation alcohol and 8% sucrose, and a fermentation temperature of 25 °C.

### 3.3. Optimization of Key Factors for Fermentation of TFG

Unlike one-step fermentation, two-step fermentation consisted of a lactic acid fermentation phase followed by an acetic acid fermentation phase. Lactic acid-fermented green tea prepared using a prolific GABA-producing strain—*L. brevis* GTL 79—and the optimized conditions for GABA production reported by Jin et al. [14] served as the fermentation material for the acetic acid fermentation phase. Optimization of fermentation conditions for the acetic acid fermentation phase of TFG was performed following the same procedure as described in the previous section. Similarly, in each optimization stage, pH, AAB counts, and contents of acetic acid were recorded. Considering that two-step fermentation entailed lactic acid fermentation, the contents of lactic acid and GABA were also analyzed. However, lactic acid content is not described in this section because the content was significantly reduced throughout the fermentation period, with increasingly severe reduction at each successive optimization stage.

**Figure 4:**
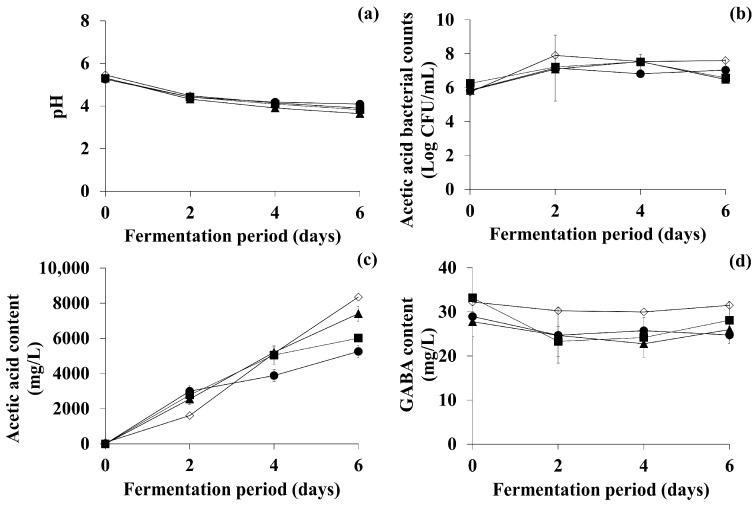
Changes in (**a**) pH, (**b**) acetic acid bacterial counts, (**c**) acetic acid content, and (**d**) GABA content in TFG at each stage of the optimization process for acetic acid fermentation. Two-step fermentation of green tea consisted of lactic acid fermentation followed by acetic acid fermentation. For lactic acid fermentation, conditions optimized in a previous study (Jin et al., 2021) [14] were used. ●: Control (fermentation without supplementation), ■: condition optimized at stage 1 (fermentation with supplementation of 3% fermentation alcohol), ▲: condition optimized at stage 2 (fermentation with supplementation of 4% sucrose in addition to the condition optimized at stage 1), ◇: condition optimized at stage 3 (fermentation at 25 °C in addition to the conditions optimized at stage 2). Sucrose was selected based on the effects of several carbohydrates at the same concentration, and the sucrose concentration was subsequently optimized. Error bars indicate standard deviations determined from triplicate experiments.

In the first optimization stage, the effects of different fermentation alcohol concentrations (1, 3, and 5%) were investigated (data not shown). When fermentation alcohol was supplemented into the acetic acid fermentation, the change patterns for pH, AAB counts, and acetic acid content were the same as in the one-step fermentation experiments. Particularly, at the end of fermentation, the TFG samples supplemented with 3% fermentation alcohol contained a significantly higher level of acetic acid (6011.83 ± 352.42 mg/L) than those of the control and the other samples (*p* < 0.05). With regard to GABA, the contents in the control and all other samples gradually decreased by day 2 of fermentation, and slightly but insignificantly increased thereafter. There was no statistically significant difference (*p* > 0.05) in the contents between all samples. Therefore, 3% fermentation alcohol supplementation was chosen as the optimal factor for acetic acid fermentation in this stage (Figure 4, ■), which was the same concentration of fermentation alcohol as in one-step fermentation.

Since sucrose was chosen as the optimal carbohydrate for acetic acid fermentation of OFG in the previous section, in the second optimization stage of acetic acid fermentation of TFG, the effects of various sucrose concentrations (0, 2, 4, 6, 8, and 10%) were examined (data not shown). When sucrose was supplemented into the acetic acid fermentation, the change patterns of pH, AAB counts, and acetic acid content were the same as in one-step fermentation. In particular, at the end of fermentation, the sucrose-supplemented samples had higher acetic acid concentrations than the control, with 4% sucrose supplementation exhibiting the highest acetic acid-producing capability (7400.28 ± 199.85 mg/L) among all of the samples (*p* < 0.05). Regarding GABA, its concentration in the control remained constant throughout fermentation. Although the GABA content in all samples gradually decreased until day 4 of fermentation, and slightly increased thereafter, the final contents in the control and all other samples were not significantly different (*p* > 0.05). Thus, in optimization stage 2 (Figure 4, ▲), 4% sucrose supplementation was eventually selected as the optimal factor, which was a lower concentration than in one-step fermentation (8% sucrose).

In the final optimization stage, the effects of fermentation at 20, 25, and 30 °C were examined (data not shown). When fermented at different temperatures, the change patterns of pH, AAB counts, and acetic acid content were similar to those in one-step fermentation. Particularly, after fermentation at 25 °C, the acetic acid content in the TFG sample was considerably higher (8349.49 ± 418.04 mg/L) than that in the other samples (*p* < 0.05). In addition, although the GABA content in all samples slightly (but not significantly) decreased until day 4 of fermentation, and then slightly (but not significantly) increased thereafter, the final contents in all samples were not significantly different (*p* > 0.05). Therefore, an optimal fermentation temperature of 25 °C was selected in this stage (Figure 4, ◇), which was the same temperature as in one-step fermentation. The changes in pH, acetic acid bacterial count, and acetic acid content obtained under the optimal conditions selected in each optimization stage are compiled in Figure 4. Altogether, the optimal fermentation conditions for the acetic acid fermentation phase of TFG were defined as supplementation with 3% fermentation alcohol and 4% sucrose, and a fermentation temperature of 25 °C.

### 3.4. Comparison of Bioactive Compounds and DPPH Scavenging Activity of OFG and TFG

To compare the effects of one- and two-step fermentation on the functionality of fermented green tea, bioactive compounds produced through the fermentation processes are summarized in Table 2. Under the optimized conditions for one- and two-step fermentation, the acetic acid contents in OFG and TFG increased 21.20- and 29.51-fold, respectively, compared to that in the control (inoculated green tea without supplementation) for one-step fermentation. Furthermore, TFG contained up to 31.49 ± 1.17 mg/L of GABA and 243.44 ± 58.15 mg/L of lactic acid, although while GABA content remained, lactic acid content decreased as acetic acid fermentation progressed. It is also noteworthy that, as shown in Table 2, the acetic acid content in each sample prepared with an optimal factor obtained from each optimization stage for the acetic acid fermentation phase of TFG was significantly higher than that in the same stage in one-step fermentation (*p* < 0.05). Thus, the acetic acid content in TFG was as high as 139.21% of that in OFG.

**Figure 5:**
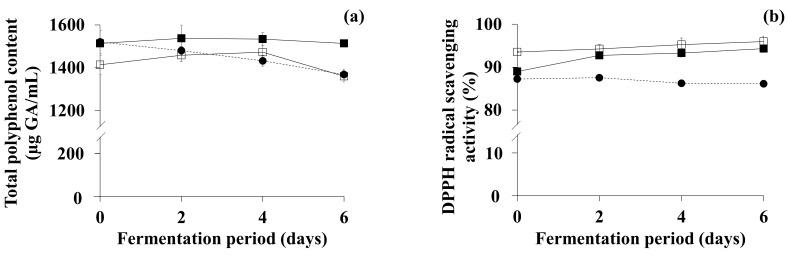
Changes in (**a**) total polyphenol content and (**b**) DPPH radical scavenging activity of OFG and TFG under each optimal fermentation condition. ●: Blank (non-inoculated green tea with no supplementation), ■: OFG (one-step fermented green tea under optimal fermentation conditions), □: TFG (two-step fermented green tea under optimal fermentation conditions). Two-step fermentation of green tea consisted of lactic acid fermentation followed by acetic acid fermentation. For lactic acid fermentation, conditions optimized in a previous study (Jin et al., 2021) [14] were used. Error bars indicate standard deviations determined from triplicate experiments.

To further evaluate the functionality of OFG and TFG under the optimized conditions, DPPH radical scavenging activity and total polyphenol content—the most representative indicators reflecting the antioxidative activity of a plant extract, as suggested by previous studies [45,46]—were determined. The change in total polyphenol content in the OFG and TFG, as well as the blank (i.e., non-inoculated green tea with no supplementation), is shown in Figure 5a. The initial total polyphenol contents of the blank and OFG were 1520.32 mg/L and 1514.11 mg/L, respectively, while that of TFG was 1414.23 mg/L. The lower initial content in TFG may have been due to a decrease in the total polyphenol content during the lactic acid fermentation phase of two-step fermentation [14]. The total polyphenol content in the blank gradually fell throughout the experimental period, while that of the OFG remained constant. The total polyphenol content in the TFG gradually increased to 1473.09 mg/L by day 4, and decreased to 1360.71 mg/L by the end of the fermentation period.

Somewhat differently from the total polyphenol content, the change in DPPH scavenging activity varied depending on the fermentation processes (Figure 5b). The initial DPPH scavenging activity of the blank and OFG was 87.25% and 88.97%, respectively, while that of TFG was 93.53%. The higher initial activity of TFG could be attributed to the bioactive compounds—including GABA, acetic acid, and lactic acid—produced through the lactic acid fermentation phase of two-step fermentation [14]. The DPPH scavenging activity of the blank slightly decreased throughout the experimental period. The DPPH scavenging activity of the OFG significantly increased to 92.74% by day 2, and slightly rose to 94.31% by the end of the fermentation period, while that of the TFG gradually increased up to 95.99% (significantly higher than the initial activity; *p* < 0.05) throughout the fermentation period. Consequently, the DPPH scavenging activity of OFG and TFG was higher (109.50% and 111.45%, respectively) than that of green tea (blank). Although the total polyphenol content in TFG was lower than that of OFG, TFG possessed significantly higher DPPH scavenging activity than OFG (*p* < 0.05), which may have been due to the higher levels of acetic acid [54], GABA [55], and lactic acid [56,57] in TFG, as shown in Table 2.

## 4. Discussion

Green tea has been described as containing a variety of bioactive compounds, such as polyphenols and catechins, that provide antioxidative, anti-obesity, and anti-carcinogenic effects [3,4,5,6,7]. To improve the health functions of green tea, several studies have tried to develop fermentation processes, including the selection of fermentation microorganisms [14,58] and optimization of fermentation conditions [14]. As part of these efforts, lactic acid fermentation with useful LAB has mainly been studied to enhance the health functions of green tea [13,14,58]. In other previous studies, acetic acid fermentation has also been studied as part of a fermentation process with yeasts to improve the health-promoting effects of kombucha—a green tea-based fermented beverage [59,60,61]. To the best of our knowledge, however, studies on two-step fermentation of green tea using both LAB and AAB have rarely been reported. Therefore, the present study was conducted to enhance acetic acid production and/or provide additional bioactive compounds to green tea through one- and two-step fermentation.

In the present study, *A. pasteurianus* PCH 325 with outstanding capability of producing acetic acid in green tea was isolated from an over-ripened peach. The fact that *A. pasteurianus* and other *Acetobacter* species—such as *A. aceti* and *A. lovaniensis*—have been reported to be commonly used in vinegar fermentation [15,16,48] was also considered in the selection of the fermentation strain. Among the nine strains selected based on the growth and acid production characteristics in the third screening stage (see Section 3.1), the six *A. okinawensis* strains were not selected after identification, because this species has not been widely used for vinegar production in the food industry. However, since a study on acetic acid production with *A. okinawensis* has been recently reported [62], further studies on the application of these strains in the food industry—such as fermentation conditions of food raw materials and safety evaluation of the strains and fermented products—will be necessary in the future. Meanwhile, several studies have reported that green tea catechins have a broad antimicrobial spectrum, to which both pathogenic bacteria and AAB are susceptible [47,63,64]. Thus, it is noteworthy that all 28 *Acetobacter* strains selected in the second screening stage showed green tea catechin resistance. According to Diez et al. [47], the resistance of AAB to catechins may be due to their ability to degrade catechins, suggesting that the level of resistance to green tea catechins of the 28 *Acetobacter* strains might be attributed to catechin degradation. Similarly, in a previous study on lactic acid-fermented green tea, while the in vitro antioxidative effect increased, the polyphenol content in green tea decreased during fermentation [14]. Taken together, bioconversion studies of bioactive compounds—such as catechins and polyphenols—in green tea using AAB as well as LAB will also be of interest to food microbiologists.

Optimization of fermentation conditions is a key process in fermentation research, along with the selection of the fermentation strain to stimulate metabolic activity and bioactive compound productivity. For acetic acid fermentation, the key factors have been intensively used in the research of fermentation process optimization [50,51,52,65,66,67]. Based on the present and previous studies, the optimized fermentation conditions vary widely even when the same species (but different strains) of *Acetobacter* are used. Since such differences may be due to the fermentation materials and the bacterial strain used, optimization of the fermentation process should be conducted when fermentation materials and/or bacterial strains are changed. To industrialize the final product, further research to increase fermentation yield through optimization of the fermentation process (such as agitation, oxygenation, temperature, pH, substrate concentrations, etc.) is necessary [68,69]. It is also worth mentioning that although most optimal key factors for one- and two-step fermentation processes were the same, the optimal sucrose concentration for two-step fermentation was lower (4%) than that for one-step fermentation (8%). These results could be explained by the possibility that several precursors of acetic acid—such as fermentation alcohol, glucose, and lactic acid, which would have been present in the lactic acid-fermented green tea—were used in the acetic acid fermentation phase of two-step fermentation. In fact, our additional experiments to confirm this possibility revealed that both the fermentation alcohol and glucose provided for lactic acid fermentation remained after the fermentation, and could be used for acetic acid fermentation (data not shown). Finally, it is also expected that the use and optimization of additional fermentation factors—such as fermentation substrates and conditions that differ from the key factors examined in this study—would further enhance the effects.

In the present study, one- and two-step fermentation showed a similar change pattern of total polyphenol contents (one of the most representative indicators reflecting the antioxidative activity of plant extracts) in OFG and TFG. While the content in TFG slightly increased until day 4 of fermentation and significantly decreased thereafter, that in OFG showed an insignificant—but similar to TFG—up-and-down change pattern. Similarly, Lee et al. [70] reported an up-and-down change pattern of the total polyphenol content in cocoa fermentation. Moreover, Teng et al. [71] reported that polyphenol oxidase in green tea leaves degraded the polyphenols in green tea, while Kong et al. [72] found that the total polyphenol content in a vinegar-like fermented papaya beverage significantly increased during fermentation. As described above, the role of microorganisms in changes in polyphenol content appears to vary; further research is necessary to elucidate the dynamic nature of microbial mechanisms involved in both production and degradation (and possibly biotransformation) of the total polyphenol content in TFG. Meanwhile, the DPPH scavenging activity of TFG was higher than that of OFG, which may be attributable to the higher levels of acetic acid, lactic acid, and GABA, as described in the previous section. Some previous studies have proven that organic acids—including acetic acid [54] and lactic acid [56]—have DPPH scavenging activity. Another previous study [52] also elucidated the role of GABA in elevating antioxidative activity by scavenging reactive oxygen species. It is also worth mentioning that acetic acid has various functionalities, such as antimicrobial [17], anti-hyperglycemic [19], antidiabetic [20], anti-hypertensive [21], hypocholesterolemic [22], anti-obesity [23], and anti-carcinogenic [24] effects, while lactic acid is known to confer an anti-inflammatory effect [73]. GABA also has health-promoting activities, including antidiabetic [74], anti-depressive [75], anti-inflammatory [76], and sleep-inductive activities [77]. Since TFG contained these bioactive compounds, and could therefore be a novel food and/or resource for providing various health-promoting activities (including antioxidative activity), it would be interesting for further research to prove its practical health functions.

Several previous studies have tried to develop two-step fermentation processes to enhance and/or provide health-promoting effects of existing or novel bioactive compounds compared to one-step fermentation [29,30,31]. In the present study, as shown in Table 2, the acetic acid content in the sample at each optimization stage of two-step fermentation was significantly higher than that in the corresponding sample of the one-step fermentation (*p* < 0.05). These results could be explained by the possibility that lactic acid—a product of lactic acid fermentation—was used as an acetic acid precursor in the acetic acid fermentation phase [78]. Alternatively, fermentation alcohol and glucose supplied as substrates during lactic acid fermentation remained and were used for acetic acid fermentation, as discussed above. Furthermore, throughout the lactic acid fermentation phase of two-step fermentation, novel functionalities of GABA and lactic acid that could not be produced in acetic acid fermentation may have been provided to the TFG (Table 2). However, considering that the content of lactic acid decreased as acetic acid fermentation progressed, it is necessary to establish an optimal fermentation period to selectively raise the yield of desired bioactive compounds in the future.

Based on the findings of the present study, two-step fermentation of green tea can not only increase the content of acetic acid, but also can supplement additional bioactive compounds, including GABA and lactic acid. Thus, compared to one-step fermentation, two-step fermentation has the potential to further enhance the health-promoting effects of green tea, and can therefore be of interest to academia and industry. In addition, it is presumed that TFG containing high levels of GABA and other organic acids would further promote human health better than OFG. It is still necessary to carry out in vivo and clinical trials to support the results of this study—obtained through in vitro tests—for further application to the clinical and food industries.

## 5. Conclusions

This study was conducted to enhance acetic acid production and/or provide additional bioactive compounds to green tea through one- and two-step fermentation processes using a prolific acetic acid-producing *A. pasteurianus* PCH 325 and optimized acetic acid fermentation conditions (and, if necessary, GABA-producing *L. brevis* GTL 79 and optimized lactic acid fermentation conditions for two-step fermentation). After applying the strain(s) and the optimized key fermentation factors, the acetic acid content in both OFG and TFG significantly increased, by 21.20- and 29.51-fold, respectively, compared to that in the control for one-step fermentation. Moreover, the acetic acid content in TFG was significantly higher (139.21%) than that in OFG. Furthermore, the lactic acid fermentation phase of two-step fermentation led to the production of GABA and lactic acid in TFG, which could have contributed to the higher DPPH scavenging activity of TFG (along with the higher content of acetic acid).

Consequently, the two-step fermentation conducted in the current study was found to provide additional bioactive compounds (i.e., GABA and lactic acid) through the lactic acid fermentation phase, as well as to significantly increase the acetic acid content throughout the acetic acid fermentation phase. Therefore, two-step fermentation may be a valuable strategy for academia and industry to enhance the functionality of green tea (or other agricultural plants). In addition, aside from the fact that bioactive compounds produced through the two-step fermentation or contained in green tea itself have their own well-known functions in improving human health, clinical research may need to be conducted in the future to determine whether OFG and TFG have significant benefits for human health.

## Figures and Tables

**Table 1:** Selection of an *Acetobacter* strain for acetic acid fermentation of green tea based on acid production, growth potential, and acetic acid production capability.

Bacterial Strains ^1^	pH	Growth Potential (OD_600_)	Green Tea Catechin Resistance		Acetic Acid Production (mg/L)
			Specific Growth Rate (h^−1^)	Relative Lag Time	
***Acetobacter* Strains Isolated From Rotten Fruits**					
*A. pasteurianus* PCH 325	3.89 ± 0.00 ^2^	1.92 ± 0.00	0.06 ± 0.00	1.11 ± 0.00	282.98 ± 25.01
Other candidate strains	3.91 ± 0.16 (3.61–4.33) ^3^	1.83 ± 0.44 (0.95–2.28)	0.07 ± 0.03 (0.01–0.12)	4.22 ± 2.36 (1.00–10.02)	0.57 ± 0.44 (0.26–0.88)
**Reference Strain of *Acetobacter* spp.**					
*A. aceti* KCTC 12290	4.45 ± 0.03	0.92 ± 0.05	0.01 ± 0.00	- ^4^	0.85 ± 0.25

^1^ Bacterial strains, including the ultimately selected *A. pasteurianus* PCH 325 strain and 27 other candidate strains selected through the first to third screening stages, were examined for acid production, growth potential, and green tea catechin resistance (the bacterial incubation was conducted at 30 °C for 72 h); bacterial strains, including the final selected strain and 2 other candidate strains selected at the fourth screening stage, were not only examined for acetic acid production capability, but also subjected to previous tests (the fermentation with each strain was conducted at 30 °C for 6 days). ^2^ Mean ± standard deviation obtained from a single strain. ^3^ Mean ± standard deviation (the range from minimum to maximum) obtained from multiple strains. ^4^ Lag phase could not be determined visually or with software.

**Table 2:** Comparison of bioactive compounds at each optimization stage for the acetic acid fermentation phase in the one- and two-step fermentation processes of green tea.

Optimization Stage (Key Factors) ^1^	One-Step Fermentation	Two-Step Fermentation		
	Acetic Acid (mg/L)	Acetic Acid (mg/L)	GABA (mg/L)	Lactic Acid (mg/L)
Control	282.98 ± 25.01 ^2,A,a^ (100.00%) ^3^	5250.00 ± 1578.00 ^A,b^ (100.00%)	24.77 ± 4.83 ^A^	243.44 ± 58.15 ^A^
First stage (fermentation alcohol)	3818.18 ± 239.56 ^B,a^ (1349.28%)	6011.83 ± 352.42 ^AB,b^ (114.51%)	28.10 ± 1.91 ^AB^	212.63 ± 80.00 ^A^
Second stage (carbohydrate)	5362.54 ± 370.70 ^C,a^ (1895.02%)	7400.28 ± 199.85 ^BC,b^ (140.96%)	25.99 ± 4.27 ^AB^	30.33 ± 3.30 ^B^
Final stage (fermentation temperature)	5997.80 ± 513.06 ^C,a^ (2119.51%)	8349.49 ± 418.04 ^C,b^ (159.04%)	31.49 ± 1.17 ^B^	88.32 ± 9.76 ^B^

^1^ Key fermentation factors applied in each optimization stage. Optimized conditions were 3% fermentation alcohol, 8% sucrose, and fermentation at 25 °C for one-step fermentation; and 3% fermentation alcohol, 4% sucrose, and fermentation at 25 °C for two-step fermentation. The inoculated green tea without supplementation served as a control. ^2^ Mean ± standard deviation of independent experiments performed in triplicate. Mean values of the contents of bioactive compounds in the same column that are followed by different upper-case letters (^A–C^) are significantly different (*p* < 0.05). Mean values of acetic acid content in the same row (one-step fermentation vs. two-step fermentation) that are followed by different lower-case letters (^a,b^) are significantly different (*p* < 0.05). ^3^ Relative acetic acid contents are represented as the percentage with respect to the corresponding control.

## Data Availability

Data is contained within this article.
